# Antihyperlipidemic Activity of *Aloe succotrina* in Rats: Possibly Mediated by Inhibition of HMG-CoA Reductase

**DOI:** 10.1155/2014/243575

**Published:** 2014-02-17

**Authors:** Dinesh Dhingra, Deepak Lamba, Ramesh Kumar, Pashupati Nath, Satyaprakash Gauttam

**Affiliations:** ^1^Department of Pharmaceutical Sciences, Guru Jambheshwar University of Science and Technology, Hisar, Haryana 125001, India; ^2^Padmnabham Ayurveda Hospital and Research Center, 75 Jawahar Nagar Colony, Tonk Road, Jaipur, Rajasthan, India; ^3^M.S.M. Institute of Ayurveda, B.P.S. Mahila Vishwavidyalaya, Khanpur Kalan, Sonipat, Haryana, India

## Abstract

The present study was designed to investigate antihyperlipidemic activity of dried pulp of *Aloe succotrina* leaves in Wistar albino rats. Hyperlipidemia was induced in rats by feeding them high fat diet (HFD) or D-fructose (25% w/v) for 4 successive weeks. From 15th to 28th day, dried pulp (100 and 200 mg/kg, p.o) and atorvastatin (10 mg/kg, p.o.) per se were administered 2 h prior to feeding rats with HFD or fructose. *Aloe succotrina* did not significantly decrease the body weight of rats. The dried pulp and atorvastatin per se significantly decreased relative liver weight but did not significantly affect relative heart weight. HFD or fructose significantly increased serum total cholesterol, triglycerides, LDL-c, and VLDL, and decreased HDL-c; significantly increased liver MDA and decreased GSH levels. The dried pulp (200 mg/kg p.o.) significantly reversed high fat diet-induced and fructose-induced hyperlipidemia and atherogenic index. *Aloe succotrina* significantly decreased HMG Co-A reductase activity. Antihyperlipidemic effect of the dried pulp was comparable to atorvastatin. Thus, *Aloe succotrina* produced significant antihyperlipidemic activity in both HFD and fructose-induced hyperlipidemic rats, possibly through normalization of serum lipid profile, HMG-CoA reductase inhibitory activity, and amelioration of oxidative stress in liver.

## 1. Introduction

Hyperlipidemia is a heterogeneous disorder commonly characterized by elevated serum total cholesterol, low density and very low-density lipoprotein cholesterol, triglycerides, and decreased high-density lipoprotein levels [1]. Hyperlipidemia is one of the greatest risk factors contributing to the prevalence and severity of atherosclerosis and subsequent coronary heart disease [2]. Liver synthesizes two-third of the total cholesterol made in the body. The rate limiting enzyme is 3-hydroxy-3-methylglutaryl (HMG)-Co A reductase and provides feedback regulation by controlling the cholesterol concentrations in cells. Treatment of hyperlipidemia involves diet control, exercise, and the use of lipid-lowering diets and drugs [3]. The most commonly employed drugs for treatment of hyperlipidemia include hydroxymethylglutarate coenzyme A (HMG-CoA) reductase inhibitors, also called as statins. Other drugs employed for treatment of hyperlipidemia include bile acid sequestrants (anion-exchange resins) such as cholestyramine and colestipol; fibrates such as clofibrate, gemfibrozil, fenofibrate, ciprofibrate, and bezafibrate; niacin; cholesterol absorption inhibitors such as ezetimibe; and omega-3-fatty acids [4].

In spite of the availability of a number of drugs for treatment of hyperlipidemia, antihyperlipidemic therapy is still deprived of the efficiency, safety, and finally “cost.” For example, there is a risk of sever muscle damage with statins, which are particularly well suited for lowering LDL [5]. Niacin, a good drug for lowering triglycerides, may cause hyperglycemia and may also cause liver damage [6]. Adverse reactions of Achilles tendon xanthomas have been reported after the addition of niacin and bile acid sequestrants to ongoing statin therapy in patients of hypercholesterolemia [7]. Adverse effects due to the use of fibrates often relate to the skeletal muscle, kidneys, or liver. Fenofibrate induced rhabdomyolysis which was complicated with acute renal failure [8]. Thus, there is still need for development of better antihyperlipidemic agents. Plant products are frequently considered to be less toxic and free from side effects than synthetic ones. There are a number of plants reported to possess antihyperlipidemic activity in clinical studies such as *Allium sativum* [9], *Commiphora mukul* [10], soy or *Glycine max* [11], *Nigella sativa* [12], and *Plantago ovata* [13].

Leaves of *Aloe succotrina *have been reported to possess antimalarial [14] and antidiabetic [15] activities. But effect of *Aloe succotrina* on hyperlipidemia has not been explored. So the aim of our study was to explore dried pulp of *Aloe succotrina* leaves on high fat diet- and fructose-induced hyperlipidemia in rats.

## 2. Materials and Methods

### 2.1. Experimental Animals

Wistar albino rats of either sex, weighing 60–100 g, were procured from Disease Free Small Animal House, Lala Lajpat Rai University of Veterinary and Animal Sciences, Hisar, Haryana. The animals were housed separately in groups of 6 per cage (43 × 27 × 15 cm) under laboratory conditions with alternating light and dark cycle of 12 h each. The animals had free access to food and water. The animals were acclimatized for at least five days before the start of experiments. The experimental protocol was approved by Institutional Animals Ethics Committee and animal care was taken as per the guidelines of Committee for the Purpose of Control and Supervision of Experiments on Animals (CPCSEA), Government. of India (Registration no. 0436).

### 2.2. Drugs and Chemicals

Atorvastatin (Ranbaxy Laboratories Ltd., Gurgaon), D-fructose, cholesterol pure, cholic acid, egg yolk powder, EDTA, n-butanol, acetic acid, tris buffer, hydrochloride, sodium lauryl sulphate, thiobarbituric acid, trichloroacetic acid, ethanol, carboxy methyl cellulose, potassium chloride, Ellman's reagent (5,5′-dithiobis-(2-nitro-benzoic acid), DTNB), pyridine, sodium hydroxide, sodium arsenate, perchloric acid, ferric chloride and hydroxyl amine hydrochloride (HiMedia Laboratories Pvt., Ltd., Mumbai) were used in the present study.

### 2.3. Plant Material

Dried pulp of *Aloe succotrina* leaves was procured from Padmnabham Ayurveda Hospital and Research Centre, Jawahar Nagar Colony, Tonk Road, Jaipur (Rajasthan, India).

### 2.4. Vehicle

Dried pulp of *Aloe succotrina* leaves and atorvastatin were separately suspended in 1% w/v CMC just before administration to the animals.

### 2.5. Induction of Hyperlipidemia

#### 2.5.1. High Fat Diet-Induced Hyperlipidemia

High fat diet was administered for 28 successive days to induce hyperlipidemia in rats [16]. High fat diet consisted of normal feed (78.8%), cholesterol pure (1%), cholic acid (0.2%), lard (10%) and egg yolk powder (10%). Normal feed contained only freshly cooked dalia (coarsely grounded wheat). Normal and high fat diets were freshly prepared each day.

#### 2.5.2. Fructose-Induced Hyperlipidemia

D-fructose (25% w/v) in drinking water was administered for 28 successive days to induce hyperlipidemia in rats [24].

### 2.6. Experimental Protocol

Animals were divided into the following groups and each group comprised of 3–6 rats.

#### 2.6.1. Groups for High Fat Diet Induced-Hyperlipidemia Model 

*Gr* *ou* *p*  1  (*n* = 6). Rats were fed with normal diet for 14 successive days. From the 15th day to the 28th day, vehicle CMC (1% w/v) was administered orally 2 h before feeding the animals with normal diet. 

*Gr* *ou* *p*  2  (*n* = 6). High fat diet was administered for 14 consecutive days. From the 15th day to the 28th day, vehicle CMC (1% w/v) was administered orally 2 h before feeding the animals with high fat diet. 

*Gr* *ou* *p*  3  (*n* = 3). High fat diet was administered for 14 consecutive days. From the 15th day to the 28th day, atorvastatin (10 mg/kg, p.o.) was administered orally 2 h before feeding the animals with high fat diet. 

*Gr* *ou* *ps*  4  *and*  5  (*n* = 5  *each*). High fat diet was administered for 14 consecutive days. From the 15th day to the 28th day, dried pulp of *Aloe succotrina* leaves (100 mg/kg and 200 mg/kg, p.o. resp.) was administered orally 2 h before feeding the animals with high fat diet.

#### 2.6.2. Groups for Fructose-Induced Hyperlipidemia Model 

*Gr* *ou* *p*  6  (*n* = 5). Rats were fed with normal diet for 14 successive days. From the 15th day to the 28th day, vehicle CMC (1% w/v) was administered orally 2 h before feeding the animals with normal diet. 

*Gr* *ou* *p*  7  (*n* = 5). Fructose (25% w/v) was administered for 14 consecutive days. From the 15th day to the 28th day, vehicle CMC (1% w/v) was administered orally 2 h before feeding the animals with fructose (25% w/v). 

*Gr* *ou* *p*  8  (*n* = 5). Fructose (25% w/v) was administered for 14 consecutive days. From the 15th day to the 28th day, atorvastatin (10 mg/kg, p.o.) was administered orally 2 h before feeding the animals with fructose solution. 

*Gr* *ou* *ps*  9  *and*  10  (*n* = 5  *each*). Fructose (25% w/v) was administered for 14 consecutive days. From the 15th day to the 28th day, dried pulp of *Aloe succotrina* leaves (100 mg/kg and 200 mg/kg, p.o., resp.) was administered orally 2 h before feeding the animals with fructose solution.

### 2.7. Measurement of Body Weight, Relative Liver, and Heart Weight of Rats

Body weights of animals of all groups were measured every week. But relative liver and heart weight of the animals were measured on the 29th day after sacrificing the animals. Relative liver and heart weight were calculated by applying the following formula [17]:

(1) $$\begin{array}{c} \text{Relative}\;\text{weight}\;=\;\left(\frac{\text{Organ}\;\text{weight}}{\text{Body}\;\text{weight}}\right)\times 100. \end{array}$$

### 2.8. Biochemical Estimations in Serum

On day 29, animals were sacrificed by carotid artery bleeding under light anesthesia with diethyl ether and blood samples (about 0.5 mL from each animal) were collected. Blood was kept for 30 min for coagulation and then serum was separated by centrifugation at 3000 rpm using centrifuge (Remi, Mumbai). Total cholesterol [18], HDL-cholesterol [19], and triglycerides [20] were measured in serum samples using commercially biochemical kits (Erba Diagnostics, Mannheim, Germany). The samples were analyzed by using semiautomatic autoanalyzer (Chem 5 plus-V_2_ autoanalyzer; Erba Mannheim, Germany).

### 2.9. Biochemical Estimations in Liver Homogenate

#### 2.9.1. Estimation of Reduced Glutathione (GSH) Levels

Reduced glutathione levels were measured in liver homogenate by using the procedure as reported earlier [21]. The tissue sample (200 mg of liver) was homogenized in 8.0 mL of 0.02 M EDTA in an ice bath. The homogenates were kept in the ice bath until used. Aliquots of 5.0 mL of the homogenates were mixed in 15.0 mL test tubes with 4.0 mL distilled water and 1.0 mL of 50% trichloroacetic acid (TCA). The tubes were centrifuged for 15 min at 3000 rpm. The supernatant (2.0 mL) was mixed with 4.0 mL of 0.4 M Tris buffer (pH 8.9), followed by addition of 0.1 mL Ellman's reagent [5,5-dithiobis-(2-nitrobenzoicacid)] and the sample was shaken. The absorbance was read within 5 min of the addition of DTNB at 412 nm against a reagent blank with no homogenate, using double beam UV-Visible spectrophotometer (Varian Cary 5000 UV-VIS-NIR Spectrophotometer, The Netherland).

#### 2.9.2. Estimation of Malondialdehyde Levels

Malondialdehyde (MDA), an index of free radical generation/lipid peroxidation, was measured in liver homogenate by using the procedure as reported earlier [22]. The tissue sample (100 mg of liver) was homogenized in 9.0 mL of KCL. Briefly, the reaction mixture consisted of 0.2 mL of 8.1% sodium lauryl sulphate, 1.5 mL of 20% acetic acid (pH 3.5), and 1.5 mL of 0.8% aqueous solution of thiobarbituric acid added to 0.2 mL of liver homogenate. The mixture was made up to 4.0 mL with distilled water and heated at 95°C for 60 min. After cooling the contents under running tap water, 5.0 mL of n-butanol and pyridine (15 : 1 v/v) and 1.0 mL of distilled water were added. The contents were centrifuged at about 4000 rpm for 10 min. The organic layer was separated out and its absorbance was measured at 532 nm using double beam UV-Visible spectrophotometer (Varian Cary 5000 UV-VIS-NIR Spectrophotometer, The Netherland) against a reagent blank. MDA values were calculated using the extinction coefficient of MDA-thiobarbituric acid complex 1.56 × 10^5^ l/mol × cm and expressed as nmol/mg tissue.

#### 2.9.3. Estimation of HMG-CoA Reductase Activity (HMG-CoA/Mevalonate Ratio)

HMG-CoA reductase activity was measured in liver homogenate using the procedure of Venugopala Rao and Ramakrishnan [23]. The ratio of HMG-CoA to mevalonate was taken as an index of enzyme activity which catalyzes the conversion of HMG to mevalonate. The lower the ratio, the higher the enzyme activity. The liver sample (100 mg) was homogenized in 1.0 mL of arsenate (1 gm/L) solution. Equal volumes (0.5 mL each) of fresh liver 10% tissue homogenate and diluted perchloric acid (50 mL/L) were mixed together. This was allowed to stand for 5 minute and centrifuged at about 2000 rpm for 10 min. This was filtered and 1 mL of filtrate was mixed with 0.5 mL of freshly prepared hydroxyl amine (2 mol/L) reagent of pH 5.5 for HMG-CoA and with 0.5 mL of freshly prepared hydroxyl amine (2 mol/L) reagent of pH 2.1 for mevalonate. After 5 minutes, 1.5 mL of ferric chloride reagent (prepared by dissolving 5.2 gm of trichloroacetic acid and 10 gm of ferric chloride in 50 mL of 0.65 mol/L HCl) was added to each of the test tube for HMG-CoA and mevalonate. The tubes were shaken well. Absorbance was read after 10 min at 540 nm versus a similarly treated arsenate blank using double beam UV-Visible spectrophotometer (Varian Cary 5000 UV-VIS-NIR Spectrophotometer, The Netherland).

### 2.10. Statistical Analysis

All values were expressed as mean ± SEM. The data were statistically analyzed using one-way ANOVA followed by Tukey-Kramer multiple comparison test. The *P* < 0.05 was considered to be statistically significant.

## 3. Results

### 3.1. Effects of Dried Pulp of *Aloe succotrina* Leaves on High Fat Diet-Induced Hyperlipidemic Rats

#### 3.1.1. Effect of *Aloe succotrina* on Body Weight

High fat diet significantly increased the body weight as compared to vehicle treated control after 2 weeks of treatment and this increase in body weight continued up to 4 weeks. Dried pulp of *Aloe succotrina* leaves (100 mg and 200 mg/kg, p.o.) and atorvastatin per se administered from the 15th day to the 28th day did not significantly decrease the body weights of high fat diet-induced hyperlipidemic rats at the 3rd and 4th weeks of treatment as compared to their respective high fat diet control (Table 1).

#### 3.1.2. Effect of *Aloe succotrina* on Relative Liver and Heart Weight

High fat diet significantly increased the relative liver weight of rats but did not significantly increase the relative heart weight of animals as compared to vehicle treated control after 28 days. Dried pulp of *Aloe succotrina* leaves (100 mg and 200 mg/kg p.o.) and atorvastatin (10 mg/kg p.o.) per se administered from the 15th day to the 28th day significantly decreased relative liver weight as compared to high fat diet control on the 29th day (Table 2) but did not significantly affect relative heart weight (Table 2).

#### 3.1.3. Effect of *Aloe succotrina* on Serum Lipid Parameters

High fat diet significantly increased serum triglycerides, total cholesterol, LDL-c, VLDL, and atherogenic index and significantly decreased HDL-cholesterol levels as compared to vehicle treated control. Dried pulp of *Aloe succotrina* leaves (200 mg/kg p.o.) and atorvastatin (10 mg/kg) per se significantly decreased serum total cholesterol, triglycerides, VLDL, LDL-c, and atherogenic index and increased HDL-cholesterol levels as compared to high fat diet control. Lower dose (100 mg/kg) of dried pulp of *Aloe succotrina* leavessignificantly decreased serum total cholesterol and LDL-c and increased HDL-cholesterol levels but did not significantly affect TG, VLDL, and atherogenic index as compared to high fat diet control (Table 3).

#### 3.1.4. Effect of *Aloe succotrina* on Liver HMG/Mevalonate Ratio, Malondialdehyde (MDA), and Reduced Glutathione (GSH) Levels

High fat diet significantly increased MDA levels and decreased GSH levels as compared to vehicle treated control. Dried pulp of *Aloe succotrina* leaves (200 mg/kg p.o.) and atorvastatin (10 mg/kg) per se significantly increased HMG/mevalonate ratio (or decreased HMG-CoA reductase activity), decreased MDA levels, and increased GSH levels as compared to high fat diet control. Lower dose (100 mg/kg) of dried pulp of *Aloe succotrina* leaves significantly decreased MDA levels and did not significantly affect GSH and HMG-CoA reductase activity as compared to high fat diet control (Table 4).

### 3.2. Effect of Dried Pulp of *Aloe succotrina* Leaves on Fructose-Induced Hyperlipidemic Rats

#### 3.2.1. Effect of *Aloe succotrina* on Body Weight

Fructose (25% w/v), dried pulp of *Aloe succotrina *leaves (100 mg and 200 mg/kg p.o.), and atorvastatin (10 mg/kg p.o.) per se did not significantly affect the body weight of rats as compared to vehicle treated control (Table 5).

#### 3.2.2. Effect of *Aloe succotrina* on Relative Liver and Heart Weight

Fructose (25% w/v) significantly increased the relative liver weight of animals but did not significantly increase the relative heart weight of animals as compared to vehicle treated control after 28 days. Dried pulp of *Aloe succotrina* leaves (100 mg and 200 mg/kg p.o.) and atorvastatin (10 mg/kg p.o.) per se significantly decreased relative liver weight of animals as compared to fructose treated control but did not significantly affect relative heart weight of animals (Table 6).

#### 3.2.3. Effect of *Aloe succotrina* on Serum Lipid Parameters

Fructose (25% w/v) significantly increased serum total cholesterol, triglycerides, VLDL, LDL-c, and atherogenic index and significantly decreased HDL-cholesterol levels as compared to vehicle treated control. Dried pulp of *Aloe succotrina* leaves (200 mg/kg p.o.) and atorvastatin (10 mg/kg p.o.) per se significantly decreased serum total cholesterol, triglycerides, VLDL, LDL-c, and atherogenic index and increased HDL-cholesterol levels as compared to fructose treated control on the 29th day. Lower dose (100 mg/kg) of the dried pulp significantly decreased serum triglycerides, VLDL, and atherogenic index but did not significantly affect total cholesterol, LDL-c, and HDL-c as compared to fructose treated control (Table 7).

#### 3.2.4. Effect of *Aloe succotrina* on Liver HMG/Mevalonate Ratio, Malondialdehyde (MDA), and Reduced Glutathione (GSH) Levels

Fructose (25% w/v) diet significantly increased MDA, and decreased GSH levels as compared to vehicle treated control. Dried pulp of *Aloe succotrina* leaves (200 mg/kg p.o.) and atorvastatin (10 mg/kg) per se significantly increased HMG/mevalonate ratio and GSH levels and decreased MDA levels as compared to fructose treated control. Lower dose (100 mg/kg) of the dried pulp significantly decreased MDA levels but did not significantly affect GSH and HMG-CoA reductase activity as compared to fructose treated control (Table 8).

## 4. Discussion

In the present study, dried pulp of *Aloe succotrina* leaves produced significant antihyperlipidemic effect in high fat diet- and fructose-induced hyperlipidemic rats. Rats fed with HFD or fructose for 4 successive weeks induced significant hyperlipidemia, as indicated by the significant increase in serum levels of TC, TG, LDL-c, and VLDL and decrease in serum levels of HDL-c. This is supported by earlier studies [17, 24, 25]. High fat diet had unfavorable effect on body weight and relative liver weight but did not significantly affect relative heart weight. This is supported by earlier study where HFD increased body weight and relative liver weight without significantly affecting relative heart weight [26]. D-fructose (25% w/v) produced increase in relative liver weight, without significantly affecting body weight and relative heart weight of animals. This result is also supported by the literature [17].

Fructose has been reported to induce hypertriglyceridemia associated with insulin resistance, hyperinsulinemia, and hypertension. After the absorption in GIT, fructose is transported *via *portal circulation to the liver, where it enters hepatocytes via the glucose transporter GLUT-5 independently of insulin and is rapidly metabolised. Fructose is metabolised into “glycerol-3-phosphate” and “acetyl CoA.” These two intermediate metabolites are then used as substrates for glycerides synthesis, contributing to VLDL-TG production in liver. The exposure of liver to such large quantities of fructose leads to rapid stimulation of lipogenesis and triglyceride accumulation which in turn contributes to reduced insulin sensitivity and hepatic insulin resistance/glucose intolerance [25, 27].

In both HFD and fructose-induced hyperlipidemia models, dried pulp of *Aloe succotrina* leaves (200 mg/kg p.o.) significantly decreased total cholesterol, triglycerides, LDL-c, VLDL, atherogenic index, and liver weight and increased HDL-c levels. Antihyperlipidemic effect of the dried pulp was comparable to atorvastatin, a standard antihyperlipidemic drug (a HMG-CoA reductase inhibitor).

Hyperlipidemia is thought to increase cholesterol content, and this in turn leads to generation of reactive oxygen species (ROS), increase in lipid peroxidation [28] and decrease in activity of reduced glutathione. It has been reported that overproduction of ROS can induce cellular damage via oxidation of critical cellular components such as membrane lipids, proteins, and DNA [29]. The increased levels of total cholesterol in blood could induce arterial endothelial dysfunction, and vascular endothelial injury is the initial factor in atherosclerosis. Therefore, lowering lipid and protecting vascular endothelium play significant roles in preventing atherosclerosis [30].

Previous studies showed that high fat diet [31] or fructose [25] enhanced hepatic oxidative damage due to hepatic stress, resulting from the burden of high fat diet or fructose metabolism. In the present study, HFD or fructose significantly increased liver MDA levels and decreased GSH levels, indicating enhancement of oxidative stress. These findings are in agreement with earlier reports [32]. Dried pulp of *Aloe succotrina* leaves (200 mg/kg) significantly increased liver GSH levels and decreased MDA levels in both HFD and fructose-induced hyperlipidemic rats, thus reduced oxidative stress.

To identify the possible mechanism of the antihyperlipidemic effect of the dried pulp more specifically, the activity of HMG-CoA reductase was determined. Cholesterol homeostasis is maintained by the two processes, namely, cholesterol biosynthesis in which HMG-CoA reductase catalyzes rate limiting process and cholesterol absorption of both dietary cholesterol and cholesterol cleared from the liver through biliary secretion [33]. The HMG-CoA/mevalonate ratio has an inverse relationship to the activity HMG-CoA reductase. The result of the present study indicated that the activity of HMG-CoA reductase is significantly inhibited by the dried pulp of *Aloe succotrina *leaves (200 mg/kg) in both HFD and fructose-induced hyperlipidemic rats.

Hyperlipidemia is a well-known risk factor for atherosclerosis, which is a major contributor to the pathogenesis of heart and vascular diseases. Elevated blood concentration of cholesterol, especially in LDL-c, constitutes the key risk factor for atherosclerosis [34]. Atherogenic index indicates the deposition of foam cells or plaque or fatty infiltration or lipids in heart, coronaries, aorta, liver, and kidneys. The higher the atherogenic index, the higher the risk of the above organs for oxidative damage [35]. *Aloe succotrina* leaves (200 mg/kg) also markedly lowered atherogenic index in both HFD and fructose-induced hyperlipidemic rats. Thus, it indicated its potential in preventing atherosclerosis.

## 5. Conclusions

Dried pulp of *Aloe succotrina* leaves produced significant antihyperlipidemic activity in both HFD and fructose-induced hyperlipidemic rats, possibly through normalization of serum lipid profile, HMG-CoA reductase inhibitory activity, and amelioration of oxidative stress in liver. Moreover, it also significantly decreased atherogenic index; thus, it might possess potential in preventing atherosclerosis. Further studies are required to isolate the active constituents involved in antihyperlipidemic activity of *Aloe succotrina*. Thus, dried pulp of *Aloe succotrina* leaves may be explored for its potential in the prevention and treatment of clinical hyperlipidemia.

## Figures and Tables

**Table 1:** Effect of dried pulp of *Aloe succotrina* leaves on body weight of HFD-induced hyperlipidemic rats.

Group number	Treatment (p.o.)	Dose (kg^−1^)	Number of animals	Body weight (g)				
				Week 0	Week 1	Week 2	Week 3	Week 4
1	Vehicle (1% w/v CMC)	10 mL	6	83.16 ± 3.98	87.66 ± 4.09	95.5 ± 3.77	103.33 ± 4.82	104.33 ± 4.78
2	Vehicle (1% w/v CMC) + HFD	10 mL	6	82.33 ± 2.96	102.16 ± 3.19^a^	112.66 ± 4.73^c^	129.66 ± 5.90^c^	136.00 ± 5.75^c^
3	Atorvastatin + HFD	10 mg	3	83.00 ± 4.44	98.16 ± 5.16	118.16 ± 8.37	124.00 ± 8.09	133.33 ± 11.86
4	*Aloe succotrina* + HFD	100 mg	5	79.16 ± 3.92	101.83 ± 5.134	124.5 ± 5.74	106.66 ± 5.48	115.8 ± 16.33
5	*Aloe succotrina* + HFD	200 mg	5	81.83 ± 5.13	104.00 ± 7.17	129.83 ± 8.25	138.83 ± 6.98	150.6 ± 7.36

In each group; values are in Mean ± SEM; data were analyzed by one-way ANOVA followed by Tukey-Kramer multiple comparison test.

F (4, 25) = 21.29; *P* < 0.0001 (for HFD treated control from week 0 to week 4).

F (4, 25) = 1.636; *P* < 0.1964 (comparison among various groups at week 1).

F (4, 25) = 4.207; *P* < 0.0097 (comparison among various groups at week 2).

F (4, 25) = 5.585; *P* < 0.0024 (comparison among various groups at week 3).

F (4, 20) = 7.901; *P* < 0.0005 (comparison among various groups at week 4).

^a^ *P* < 0.05 and ^c^ *P* < 0.001 as compared to HFD treated control (week 0).

**Table 2:** Effect of dried pulp of *Aloe succotrina* leaves on relative liver and heart weight of HFD-induced hyperlipidemic rats.

Group number	Treatment (p.o.)	Dose (kg^−1^)	Number of animals	Relative organ weight	
				Liver weight	Heart weight
1	Vehicle (1% w/v CMC)	10 mL	6	4.98 ± 0.32	0.63 ± 0.07
2	Vehicle (1% w/v CMC) + HFD	10 mL	6	7.80 ± 0.54^###^	0.70 ± 0.04
3	Atorvastatin + HFD	10 mg	3	5.77 ± 0.31^a^	0.59 ± 0.06
4	*Aloe succotrina* + HFD	100 mg	5	6.87 ± 0.32	0.64 ± 0.04
5	*Aloe succotrina* + HFD	200 mg	5	5.81 ± 0.34^a^	0.60 ± 0.02

Relative weight = (organ weight/body weight) × 100. Values are in Mean ± SEM; data were analyzed by one-way ANOVA followed by Tukey-Kramer multiple comparison test.

F (4, 20) = 8.271; *P* < 0.0004 (for relative liver weight).

F (4, 20) = 0.747; *P* < 0.5715 (for relative heart weight).

^###^ *P* < 0.001 as compared to vehicle treated control; ^a^ *P* < 0.05 as compared to HFD treated control.

**Table 3:** Effect of dried pulp of *Aloe succotrina* leaves on serum lipid levels in HFD-induced hyperlipidemic rats.

Group number	Treatment (p.o.)	Dose (kg^−1^)	Lipid levels (mg/dL)					
			TC	TG	HDL-c	VLDL	LDL-c	AI (TG/HDL)
1	Vehicle (1% w/v CMC) (*n* = 6)	10 mL	78.24 ± 4.08	19.55 ± 5.04	37.18 ± 0.45	3.91 ± 1.01	36.99 ± 4.64	0.28 ± 0.07
2	Vehicle (1% w/v CMC) + HFD (*n* = 6)	10 mL	315.42 ± 27.17^###^	108.04 ± 11.67^###^	17.38 ± 3.97^###^	21.60 ± 2.34^###^	276.40 ± 26.38^###^	10.57 ± 3.86^#^
3	*Atorvastatin* + HFD (*n* = 3)	10 mg	156.06 ± 20.65^c^	28.42 ± 6.65^b^	50.37 ± 3.67^c^	5.68 ± 1.33^b^	100.00 ± 20.36^c^	0.55 ± 0.09^a^
4	*Aloe succotrina* + HFD (*n* = 5)	100 mg	231.32 ± 22.00^a^	94.90 ± 21.38	33.94 ± 3.30^b^	18.98 ± 4.28	178.45 ± 22.40^b^	2.84 ± 0.63
5	*Aloe succotrina* + HFD (*n* = 5)	200 mg	181.30 ± 12.26^c^	48.34 ± 9.66^a^	47.65 ± 1.24^c^	9.67 ± 1.93^a^	123.98 ± 11.70^c^	1.02 ± 0.22^a^

Values are in Mean ± SEM; data was analyzed by one-way ANOVA followed by Tukey-Kramer multiple comparison test. TC—total cholesterol; TG—triglycerides; HDL-c—high density lipoprotein-cholesterol; LDL-c—low density lipoprotein-cholesterol; VLDL—very low density lipoprotein- cholesterol; AI—atherogenic index.

F (4, 20) = 25.856; *P* < 0.0001 (for total cholesterol).

F (4, 20) = 10.308; *P* < 0.0001 (for triglycerides).

F (4, 20) = 21.007; *P* < 0.0001 (for HDL-c).

F (4, 20) = 10.297; *P* < 0.0001 (for VLDL).

F (4, 20) = 27.085; *P* < 0.0001 (for LDL-c).

F (4, 20) = 4.623; *P* < 0.0083 (for AI).

^#^ *P* < 0.05 and ^###^ *P* < 0.001 as compared to vehicle treated control; ^a^ *P* < 0.05, ^b^ *P* < 0.01, and ^c^ *P* < 0.001 as compared to HFD treated control.

**Table 4:** Effect of dried pulp of *Aloe succotrina* leaves on liver HMG/mevalonate ratio, malondialdehyde (MDA), and reduced glutathione (GSH) levels of HFD-induced hyperlipidemic rats.

Group number	Treatment (p.o.)	Dose (kg^−1^)	Number of animals	HMG/mevalonate ratio	MDA levels (nmol/mg tissue)	GSH levels (*μ*mol/mg tissue)
1	Vehicle (1% w/v CMC)	10 mL	6	0.59 ± 0.23	52.60 ± 10.49	735.21 ± 57.31
2	Vehicle (1% w/v CMC) + HFD	10 mL	6	0.37 ± 0.13	179.74 ± 23.77^##^	297.62 ± 24.81^###^
3	Atorvastatin + HFD	10 mg	3	3.29 ± 0.37^c^	13.43 ± 4.65^c^	665.84 ± 24.34^c^
4	*Aloe succotrina *+ HFD	100 mg	5	0.81 ± 0.13	78.56 ± 27.66^b^	303.84 ± 31.85
5	*Aloe succotrina *+ HFD	200 mg	5	1.68 ± 0.34^b^	52.72 ± 12.32^c^	617.86 ± 26.35^c^

Values are in Mean ± SEM; data was analyzed by one-way ANOVA followed by Tukey-Kramer multiple comparison test.

F (4, 20) = 14.402; *P* < 0.0001 (for HMG/mevalonate ratio).

F (4, 20) = 10.117; *P* < 0.0001 (for MDA levels).

F (4, 20) = 25.390; *P* < 0.0001 (for reduced glutathione levels).

^##^ *P* < 0.01 and ^###^ *P* < 0.001 as compared to vehicle treated control; ^b^ *P* < 0.05 and ^c^ *P* < 0.001 as compared to HFD treated control.

**Table 5:** Effect of dried pulp of *Aloe succotrina* leaves on body weight of fructose-induced hyperlipidemic rats.

Group number	Treatment (p.o.)	Dose (kg^−1^)	Body weight (g)				
			Week 0	Week 1	Week 2	Week 3
1	Vehicle (1% w/v CMC)0	10 mL	80.66 ± 4.39	83.83 ± 5.43	94.00 ± 4.97	102.33 ± 4.69	114.16 ± 5.52
2	Vehicle (1% w/v CMC) + fructose (25% w/v)	10 mL	81.16 ± 7.35	88.16 ± 9.00	92.00 ± 10.34	95.16 ± 10.42	106.83 ± 11.08
3	Atorvastatin + fructose (25% w/v)	10 mg	78.66 ± 3.54	86.83 ± 4.33	95.66 ± 4.75	102.83 ± 3.52	113.16 ± 3.09
4	*Aloe succotrina *+ fructose (25% w/v)	100 mg	83.16 ± 5.40	91.66 ± 7.19	99.83 ± 7.04	96.83 ± 6.56	106.8 ± 9.50
5	*Aloe succotrina* + fructose (25% w/v)	200 mg	80.83 ± 4.47	86.5 ± 6.13	96.16 ± 5.50	106.00 ± 4.44	119.16 ± 6.14

(*n* = 5) in each group. Values are in Mean ± SEM; data was analyzed by one-way ANOVA followed by Tukey-Kramer multiple comparison test.

F (4, 25) = 0.95; *P* < 0.04516 (for fructose treated control from week 0 to week 4).

F (4, 25) = 0.19; *P* < 0.9431 (comparison among various groups at week 1).

F (4, 25) = 0.18; *P* < 0.9464 (comparison among various groups at week 2).

F (4, 25) = 0.49; *P* < 0.7422 (comparison among various groups at week 3).

F (4, 25) = 0.49; *P* < 0.7432 (comparison among various groups at week 4).

**Table 6:** Effect of dried pulp of *Aloe succotrina* leaves on relative liver and heart weight of fructose-induced hyperlipidemic rats.

Group number	Treatment (p.o.)	Dose (kg^−1^)	Relative organ weight	
			Liver weight
1	Vehicle (1% w/v CMC)	10 mL	3.48 ± 0.15	0.40 ± 0.01
2	Vehicle (1% w/v CMC) + fructose (25% w/v)	10 mL	5.78 ± 0.74^##^	0.42 ± 0.02
3	Atorvastatin + fructose (25% w/v)	10 mg	3.87 ± 0.36^a^	0.47 ± 0.03
4	*Aloe succotrina *+ fructose (25% w/v)	100 mg	3.92 ± 0.30^a^	0.46 ± 0.02
5	*Aloe succotrina *+ fructose (25% w/v)	200 mg	3.87 ± 0.29^a^	0.49 ± 0.03

Relative weight = (organ weight/body weight) × 100.

(*n* = 5) in each group. Values are in Mean ± SEM; data was analyzed by one-way ANOVA followed by Tukey-Kramer multiple comparison test.

F (4, 21) = 4.742; *P* < 0.007 (for relative liver weight).

F (4, 21) = 1.594; *P* < 0.1045 (for relative heart weight).

^##^ *P* < 0.01 as compared to vehicle treated control; ^a^ *P* < 0.05 as compared to fructose treated control.

**Table 7:** Effect of the dried pulp of *Aloe succotrina *leaves on serum lipid levels in fructose-induced hyperlipidemic rats.

Group number	Treatment (p.o.)	Dose (kg^−1^)	Lipid Levels (mg/dL)					
			TC	TG	HDL-c	VLDL	LDL-c	AI (TG/HDL)
1	Vehicle (1% w/v CMC)	10 mL	67.09 ± 2.74	39.47 ± 1.75	37.26 ± 1.83	7.93 ± 0.37	21.83 ± 4.54	1.07 ± 0.06
2	Vehicle (1% w/v CMC) + fructose (25% w/v)	10 mL	85.56 ± 4.37^#^	67.10 ± 12.69^#^	17.36 ± 1.49^#^	13.42 ± 2.54^#^	54.80 ± 6.03^##^	3.93 ± 0.74^###^
3	Atorvastatin + fructose (25% w/v)	10 mg	64.32 ± 4.09^a^	22.59 ± 2.14^c^	41.72 ± 6.06^b^	4.52 ± 0.43^c^	18.09 ± 4.89^b^	0.60 ± 0.10^c^
4	*Aloe succotrina *+ fructose (25% w/v)	100 mg	71.97 ± 6.12	33.22 ± 3.98^b^	42.33 ± 5.48	6.64 ± 0.80^b^	38.53 ± 6.94	1.42 ± 0.32^c^
5	*Aloe succotrina *+ fructose (25% w/v)	200 mg	66.15 ± 2.16^a^	24.74 ± 2.44^c^	26.80 ± 4.71^b^	4.95 ± 0.49^c^	18.87 ± 5.27^b^	0.62 ± 0.09^c^

(*n* = 5) in each group; values are in Mean ± SEM; data was analyzed by one-way ANOVA followed by Tukey-Kramer multiple comparison test.

TC—total cholesterol; TG—triglycerides; HDL-c—high density lipoprotein- cholesterol.

LDL-c—low density lipoprotein- cholesterol; VLDL—very low density lipoprotein- cholesterol; AI—atherogenic index.

F (4, 21) = 4.344; *P* < 0.0109 (for total cholesterol); F (4, 21) = 8.425; *P* < 0.0004 (for triglycerides); F (4, 21) = 6.170; *P* < 0.0021 (for HDL-c); F (4, 21) = 8.414; *P* < 0.0004 (for VLDL); F (4, 21) = 8.118; *P* < 0.0005 (for LDL-c); F (4, 21) =14.361; *P* < 0.0001 (for AI).

^#^ *P* < 0.05, ^##^ *P* < 0.01, and ^###^ *P* < 0.001 as compared to vehicle treated control; ^a^ *P* < 0.05, ^b^ *P* < 0.01, and ^c^ *P* < 0.001 as compared to fructose treated control.

**Table 8:** Effect of dried pulp of *Aloe succotrina* leaves on liver HMG/mevalonate ratio, MDA, and reduced glutathione (GSH) levels of fructose-induced hyperlipidemic rats.

Group number	Treatment (p.o.)	Dose (kg^−1^)	HMG/mevalonate ratio	MDA levels (nmol/mg tissue)	GSH levels (*μ*mol/mg tissue)
1	Vehicle (1% w/v CMC)	10 mL	3.44 ± 1.22	7.64 ± 1.56	524.55 ± 35.89
2	Vehicle (1% w/v CMC) + fructose (25% w/v)	10 mL	1.98 ± 0.36	49.02 ± 0.92^###^	317.20 ± 19.40^###^
3	Atorvastatin + fructose (25% w/v)	10 mg	6.17 ± 0.93^a^	6.92 ± 0.69^c^	445.74 ± 19.57^a^
4	*Aloe succotrina* + fructose (25% w/v)	100 mg	3.75 ± 0.78	31.66 ± 8.33^a^	324.04 ± 13.31
5	*Aloe succotrina* + fructose (25% w/v)	200 mg	5.66 ± 0.67^a^	8.85 ± 2.05^c^	428.13 ± 34.29^a^

(*n* = 5) in each group; values are in Mean ± SEM; data was analyzed by one-way ANOVA followed by Tukey-Kramer multiple comparison test.

F (4, 21) = 4.140; *P* < 0.0001 (for HMG/mevalonate ratio).

F (4, 21) = 7.607; *P* < 0.0007 (for MDA levels).

F (4, 21) = 11.289; *P* < 0.0001 (for reduced glutathione).

^###^ *P* < 0.001 as compared to vehicle treated control.

^a^ *P* < 0.05, ^b^ *P* < 0.05, and ^c^ *P* < 0.001 as compared to fructose treated control.
